# The Effects of Moderate Physical Exercise on Adult Cognition: A Systematic Review

**DOI:** 10.3389/fphys.2018.00667

**Published:** 2018-06-08

**Authors:** Rafael M. Fernandes, Marcio G. Correa, Marcio A. R. dos Santos, Anna P. C. P. S. C. Almeida, Nathália C. F. Fagundes, Lucianne C. Maia, Rafael R. Lima

**Affiliations:** ^1^Laboratory of Functional and Structural Biology, Institute of Biological Sciences, Federal University of Pará, Belém, Brazil; ^2^Nucleus of Transdisciplinary Studies in Basic Education, Federal University of Pará, Belém, Brazil; ^3^Pediatric Dentistry and Orthodontics, Federal University of Rio de Janeiro, Rio de Janeiro, Brazil

**Keywords:** moderate physical exercise, cognition, reaction time, physical activity, physical exercise

## Abstract

**Background:** Physical exercise is a systematic sequence of movements executed with a predefined purpose. This muscular activity impacts not only on circulatory adaptations, but also neuronal integration with the potential to influence cognition. The aim of this review was to determine whether the literature supports the idea that physical exercise promotes cognitive benefits in healthy adults.

**Methods:** A systematic search for relevant articles was performed according to the Preferred Reporting Items for Systematic Review and Meta-Analysis criteria using available databases (PubMed, LILACS, Scopus, Web of Science, The Cochrane Library, OpenGrey, Google Scholar and CENTRAL). The search terms included “humans” or “adults” or “cognition” or “awareness” or “cognitive dissonance” or “cognitive reserve” or “comprehension” or “consciousness” and “motor activity” or “exercise” or “physical fitness,” and not “aged” or “nervous system diseases,” with the purpose of finding associations between moderate physical exercise and cognition. A methodological quality and risk of bias unit assessed the eligibility of articles.

**Results:** A total of 7179 articles were identified. Following review and quality assessment, three articles were identified to fulfill the inclusion criteria. An association between moderate physical exercise and cognition was observed. Improvements in cognitive parameters such as reduced simple reaction time, improved response precision and working memory were identified among the included articles.

**Conclusion:** This systematic review found that moderate physical exercise improves cognition.

## Introduction

Physical exercise consists of a systematic sequence of movements executed with a predefined purpose (American College of Sports Medicine, 2009; Bowden et al., 2011) and is described by its effect on oxygen consumption as a percentage of maximum oxygen consumption (VO_2_ max) (Barstow et al., 1990; Drummond et al., 2005). The concept of physical exercise is a subcategory of physical activity (Caspersen et al., 1985). Since the seminal paper by Morris and Crawford (1958) which evaluated health in bus drivers, several papers have reported an association between regular exercise activity and a reduced risk for the development of cancer and cardiovascular disease. However, its effect on brain function seems less explored.

Neuronal integration involves multiple cognitive functions and neuronal networks, including work memory and spatial memory (Tucker and Stern, 2011; Moon et al., 2017). Both neurons and astrocytes need a constant supply of nutrients (glucose and lactate) and oxygen, and regional cerebral oxygenation may be limited during exercise (Nielsen et al., 1999; Rooks et al., 2010; Braz and Fisher, 2016) with a change in the balance of cerebraI metabolism (Avola et al., 2004; Dalsgaard and Secher, 2007; van Hall et al., 2009). In addition, specific neurotrophic growth factors seem to be released as a result of moderate physical exercise, which increases the expression of brain-derived neurotrophic factor (BDNF) and neuronal growth factor (NGF) (Tyler and Pozzo-Miller, 2003; Dietrich et al., 2008; Seifert et al., 2010; Bonini et al., 2013; Coelho et al., 2014; Hashimoto et al., 2018). These factors contribute to increased cell survival and differentiation, as well as resistance to oxidative stress (Dietrich et al., 2008; Coelho et al., 2014). Furthermore, they can modulate neuroplasticity with an effect on brain function (Erickson et al., 2013). Another possible brain mechanism mediated by physical exercise could be related to the orexin system, increasing the release of orexin and orexin b. These are neuropeptides that modulate synaptic plasticity, neurogenesis, and cognition (Chieffi et al., 2017b,c).

Physical exercise may influence an individual's cognitive ability through its integrative effect on circulation and cerebral activity (van den Berg et al., 2016; Best et al., 2017); however, the scientific literature on this topic is limited. This systematic review aims to find evidence pointing to the effects of moderate physical exercise on cognitive functions in healthy adults.

## Materials and methods

### Study design

A systematic review was conducted according to the Preferred Reporting Items for Systematic Review and Meta-Analysis (PRISMA) guidelines (Moher et al., 2009). The review was registered in PROSPERO, the international prospective register of systematic reviews (CRD42016049663).

### Participants, interventions, and comparators

This research followed the PICO strategy, as recommended by PRISMA group (Moher et al., 2009), to develop the search criteria and determine which relevant articles to include or exclude. Interventional studies focused on the effects of moderate physical exercise on healthy adults were included, presented in Table 1.

**Table 1:** Inclusion criteria following the PICOS strategy (Moher et al., 2009).

**Inclusion Criteria**	
P (participants)	Healthy humans at any age
I (intervention)	Physical activity
C (comparison)	Absence of Physical activity
O (Outcome)	Primary outcome: Changes is cognition
	Secondary outcome: Correlation or association of another aspects, such as age, gender, previous history of smoking, alcohol, and body mass index in cognition outcome
S (type of studies included)	Studies of intervention

### Systematic review protocol

Two evaluators (MGC and RMF) performed an independent search, evaluated the validity of the publications and extracted duplicates of documents. The studies were evaluated based on the relevance of their titles and abstracts, and those that did not meet the PICO criteria were excluded. The evaluators then read the full-text versions of the selected articles. In case of disagreement, the two authors would give a joint evaluation. If any doubt remained, a third member of the research team was consulted (NCFF).

All articles that met the study requirements were included in this review. The exclusion criteria included *in vitro* experiments, literature reviews, laboratory animal studies, letters to the editor, case reports, opinions and guides. Studies that did not include cognitive tests were also excluded.

### Search strategy

Electronic searches were conducted in eight databases: PubMed, LILACS, Scopus, Web of Science, The Cochrane Library, OpenGrey, Google Scholar, The Cochrane Library and the The Cochrane Central Register of Controlled Trials (CENTRAL). Studies that investigated the effect of physical activity on the cognition of adult individuals without any central nervous system disorders or diseases (Supplementary Material) were included.

At the end of the search, alerts were created in each of the databases to identify any further references that could be included in this study. We also searched the references in the selected articles to find articles that met the search categories but had not been found in the selected databases. All reference records were imported to a reference manager software (EndNote X7, Thomas Reuters, Philadelphia, USA).

### Selection of studies and data extraction

After the importation of the searches, the duplicated results were removed. The selection process was performed in two phases. The first phase includes an evaluation of tittles and abstracts according to PICO strategy (Table 1). In the phase two, the remaining articles were evaluated by full text according to the same criteria. The searches and selection process were conducted by two examiners (MGC and RMF) and checked by a third examiner (NCFF), in cases of disagreements.

After this phase, the data extraction of the included studies was performed. The data regarding the type of study, objective, sample characteristics, physical training, cognitive test, and main results were included in a table. In case of absence of information that makes data extraction or risk of bias evaluation impracticable, we attempted to contact the authors by e-mail. The contact consisted in sending a weekly email, for up to five consecutive weeks.

### Risk of bias

The same evaluators also independently analyzed the methodological quality of all articles. Randomized trials were evaluated according to the Cochrane Collaboration's Risk of Bias tool. This tool evaluates the risk of bias as “low,” “high,” or “unclear” (Higgins et al., 2011) according to the items described in Table 2.

**Table 2:** Criteria for risk assessment of bias according to “The Cochrane Collaboration's tool for assessing risk of bias (Higgins et al., 2011).

**RANDOM SEQUENCE GENERATION**	
Criteria for judgment of “Low risk” of bias	The articles that appropriately described the method of randomization
Criteria for judgment of “High risk” of bias	Articles that presented a methodological failure in the randomization criterion or the difficult reproducibility method
Criteria for judgment of “Unclear” of bias	When the articles did not describe the method of randomization
**ALLOCATION CONCEALMENT**	
Criteria for judgment of “Low risk” of bias	When the allocation sequence of samples were concealed in the randomization
Criteria for judgment of “High risk” of bias	When the sequence of allocation of samples were not concealed at randomization
Criteria for judgment of “Unclear risk” of bias	When the allocation sequences were unreported
**BLINDING OF PARTICIPANTS AND RESEARCHERS**	
Criteria for judgment of “Low risk” of bias	When the sample was blind
Criteria for judgment of “High risk” of bias	If the methodology could not be blinded for whatever reason (sample/appraiser)
Criteria for judgment of “Unclear risk” of bias	When the sample was not reported either way
**BLINDING OF OUTCOME ASSESSMENT**	
Criteria for judgment of “Low risk” of bias	When the evaluators reported that the blinding in the evaluation was effective
Criteria for judgment of “High risk” of bias	If the study informed the evaluators how the blinding was done
Criteria for judgment of “Unclear risk” of bias	When the blinding was not reported
**INCOMPLETE OUTCOME DATA**	
Criteria for judgment of “Low risk” of bias	When there was an exhaustive description of the main data
Criteria for judgment of “High risk” of bias	If there was a loss due to an incomplete description of the main results regardless of quantity, nature and manipulation
Criteria for judgment of “Unclear risk” of bias	When the results were not reported
**SELECTIVE REPORTING**	
Criteria for judgment of “Low risk” of bias	When the discussion excluded some of the results
Criteria for judgment of “High risk” of bias	When the article discussed the data completely
Criteria for judgment of “Unclear risk” of bias	When the organization of the results in the discussion were unclear

For non-randomized studies, the Methodological Index for Non-Randomized Studies (MINORS) was used (Slim et al., 2003). This ranked the articles according to a list of 12 items, and each reviewer scored the articles according to a standard checklist where a score of 0 represented “not reported,” 1 was “reported but inadequate” and 2 was “reported and suitable” for the items described in Table 3.

**Table 3:** Criteria for risk assessment of bias according to Methodological Index for Non-Randomized Studies (MINORS) (Slim et al., 2003).

**CLEARLY STATED AIM**	
Criteria for judgment of “0” of bias	When the objectives were not expressed
Criteria for judgment of “1” of bias	When a clear relationship between the objectives, results and conclusion was not found
Criteria for judgment of “2” of bias	When there was a relationship between the objectives of the article and its results and conclusions
**INCLUSION OF CONSECUTIVE PATIENTS**	
Criteria for judgment of “0” of bias	When the objectives were not expressed
Criteria for judgment of “1” of bias	When only the inclusion criteria was described or when it was not clear how the sample was selected
Criteria for judgment of “2” of bias	When the terms led to the samples' inclusion and exclusion and were clearly described
**PROSPECTIVE DATA COLLECTION**	
Criteria for judgment of “0” of bias	When there was no information reported
Criteria for judgment of “1” of bias	When there were changes
Criteria for judgment of “2” of bias	When the data collection was already established at the beginning of the study
**ENDPOINTS APPROPRIATE TO STUDY AIM**	
Criteria for judgment of “0” of bias	When unreported
Criteria for judgment of “1” of bias	If the techniques were not well explained or leave doubt regarding the methodology
Criteria for judgment of “2” of bias	If the evaluation techniques are well explained and were already referenced and answered the purpose of the study
**UNBIASED ASSESSMENT OF STUDY ENDPOINT**	
Criteria for judgment of “0” of bias	When it was not reported
Criteria for judgment of “1” of bias	When, due to some limitation, the methodology was not a blind sample
Criteria for judgment of “2” of bias	If the existence of the double-blind analysis was described
**FOLLOW-UP PERIOD APPROPRIATE TO STUDY AIM**	
Criteria for judgment of “0” of bias	When the results were unreported
Criteria for judgment of “1” of bias	When the literature did not provide information about the training time and the post-training window
Criteria for judgment of “2” of bias	If the literature referenced the training time preference and the post-training window
**LOSS TO FOLLOW UP <5%**	
Criteria for judgment of “0” of bias	When the results were not reported
Criteria for judgment of “1” of bias	When the loss was higher than 5% and justified by the study
Criteria for judgment of “2” of bias	When the loss in the sample was reported as <5%
**PROSPECTIVE CALCULATION OF STUDY SIZE**	
Criteria for judgment of “0” of bias	When the results were not reported
Criteria for judgment of “1” of bias	When the sample calculation was not performed, but explained
Criteria for judgment of “2” of bias	When the calculation methodology for the definition of the sample was reported
**AN ADEQUATE CONTROL GROUP**	
Criteria for judgment of “0” of bias	When the criteria was not reported
Criteria for judgment of “1” of bias	When the criteria for group choice were unclear
Criteria for judgment of “2” of bias	If the control group was made up of sedentary, healthy people and the criteria for the group selection was clear
**CONTEMPORARY GROUPS**	
Criteria for judgment of “0” of bias	When these details were not reported
Criteria for judgment of “1” of bias	When one of the groups already had a training routine
Criteria for judgment of “2” of bias	When the groups started the training together
**BASELINE EQUIVALENCE OF GROUPS**	
Criteria for judgment of “0” of bias	When these details were not reported
Criteria for judgment of “1” of bias	When the groups did not have the same demographic characteristics
Criteria for judgment of “2” of bias	When the groups had similar demographics
**ADEQUATE STATISTICAL ANALYSES**	
Criteria for judgment of “0” of bias	When the tests were either unsuitable for the type of study or not reported
Criteria for judgment of “1” of bias	When the descriptions of the performed tests were not clear
Criteria for judgment of “2” of bias	When the test was used according to the type of study and was clearly described

The quality of the article was evaluated by the sum of the values obtained in the MINORS qualifier, with a score of 0–6 indicating very low quality, 7–12 low quality, 13–18 moderate quality and 19–24 representing high quality (Khan et al., 2015).

## Results

### Description of studies

**Figure 1:**
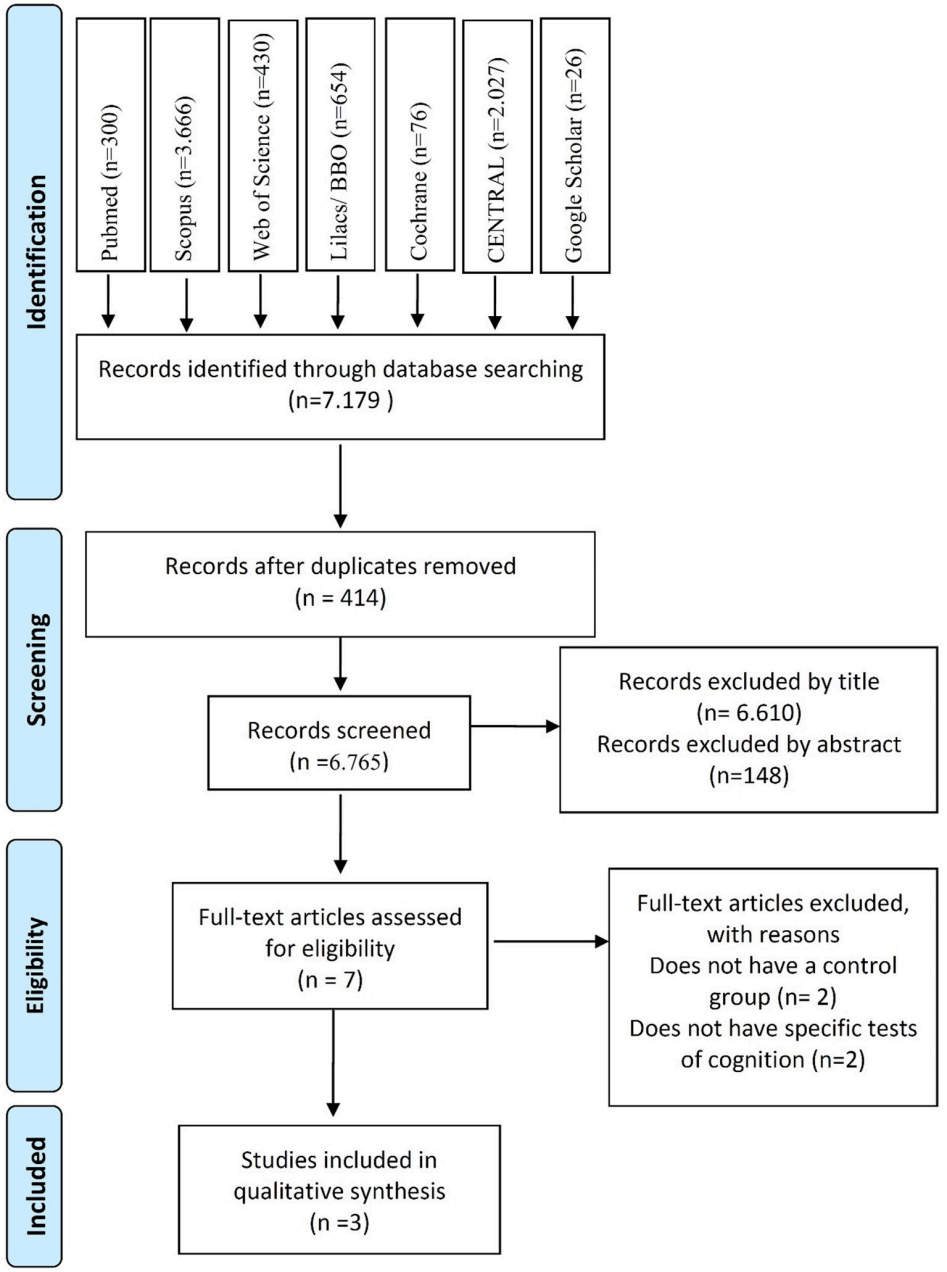
Flow diagram of the literature search according to items in the Preferred Reporting Items for Systematic Review and Meta-Analysis (PRISMA) guideline.

There were a total of 7,179 citations about this topic identified in the different databases. Scopus had the most (*n* = 3,666), followed by Central Register of Controlled-CENTRAL (*n* = 2,027), Lilacs (*n* = 654), Web of Science (*n* = 430), PubMed (*n* = 300), Cochrane Library (*n* = 76), Google Scholar (*n* = 26) then finally OpenGrey (*n* = 0). Figure 1 summarizes the study selection process. From the 7,179 citations, 414 duplicates were withdrawn and 6,765 remained.

A total of 155 papers were selected according to their title and abstract, of which seven (Biddle and Ashford, 1988; Brown et al., 1995; Bue-Estes et al., 2008; Douw et al., 2014; Lee et al., 2014; Tsai et al., 2014; Olson et al., 2016) were chosen based on the eligibility criteria for full-text reading. Four articles were excluded, two (Douw et al., 2014; Lee et al., 2014) were excluded due to the absence of a control group, and the other two (Biddle and Ashford, 1988; Brown et al., 1995) due to the absence of specific cognition tests. Three articles (Bue-Estes et al., 2008; Tsai et al., 2014; Olson et al., 2016) were selected and submitted to a qualitative synthesis.

### Study selection and characteristics

The main characteristics of the included articles are listed in Table 4. In the three studies there was an association between moderate physical exercise and cognition. While the included articles each used a different evaluation tool, they all evaluated reaction time as a common parameter to measure cognition. Reaction time is the time elapsed between a stimuli and the response. Each study showed a positive correlation between moderate physical exercise by a different parameter improvement as tested by the following methods: Automated neuropsychological assessment metrics, which evaluates the reaction time, memory, attention, concentration, thinking speed and decision making; the Eriksen flanker task test, which evaluated the reaction time and precision of the response; Visuospatial attention, which assess the reaction time.

**Table 4:** Data extraction from selected studies.

	**Type of study**	**Objective of the study**	**Sample**	**Type of training**	**Cognitive test**	**Statistical analysis**	**Results**
Bue-Estes et al., 2008	Analytical-experimental, Non-randomized study	To analyse the effect of acute and chronic aerobic exercise on the cognitive performance of adult women	27 Women between the ages of 18 and 25	Treadmill in the intensities 25, 50, 75, and 100% of VO_2_ max.	Automated neuropsychological assessment metrics (ANAM)	Linear regression analysis	In this study, the trained group showed improvement in the Simple Reaction test, with shorter reaction times, as well as improved working memory in relation to the sedentary group SIMPLE REACTION: The following result shows the response time of individuals in milliseconds (ms). sedentary (285 ms, SD 49.3) and active (252 ms SD 27.4), *p* < 0.009 WORKING MEMORY: *p* < 0.001
Olson et al., 2016	Analytical-experimental study, randomized	To investigate the dose-response effects of aerobic exercise intensity on cognitive control through behavioral and neuroelectric analysis	27 Adults 18–35 years old (men and women)	Bicycle ergometer group low intensity 40% and moderate intensity 60% of VO_2_ max.	Eriksen flanker task test	Analysis of Variance (Anova) *post hoc* Bonferroni	The results of this study show a significant difference for moderate intensity in relation to low and control in the test of reaction time and reduction in the accuracy of response REACTION TIME: *p* < 0.05 (congruent and incongruent) Accuracy of response: *p* < 0.05 (incongruent)
Tsai et al., 2014	Analytical-experimental study, randomized	To evaluate the effect of aerobic exercise of moderate intensity on the behavioral, neuroelectric performance in the BDNF levels, and a possible correlation between these factors	60 Men between the ages of 19 and 28	Moderate group VO_2_ max. = 58.04–6.67 mL/kg/min and low group VO_2_ max. = 36.04–3.64 mL/kg/min)	Visuospatial Attention Test	Analysis of Variance (Anova) *post hoc* Bonferroni	In this work the low intensity group was better than control and the moderate intensity was superior to the two groups, in the reaction time test and, without difference in the accuracy rate REACTION TIME: *p* < 0.05

### Risk of bias

**Figure 2:**
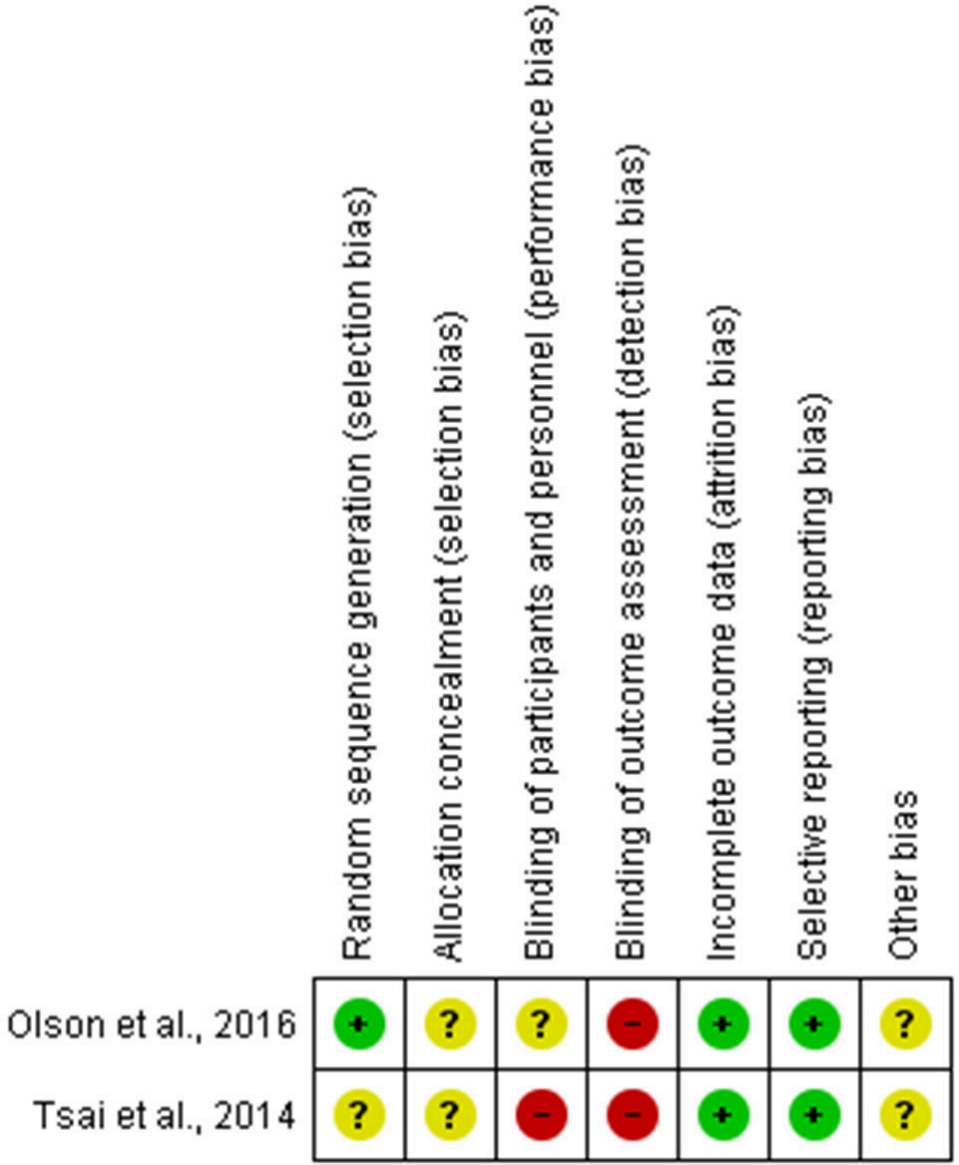
Cochrane Collaboration's tool for assessing risk of bias (adapted from Higgins and Altman).

The risk of bias in randomized articles was determined based on the Cochrane Collaboration's tool (Figure 2). When evaluated, the “blinding of outcome assessment” was classified as high risk in two studies. For the topics “incomplete outcome data” and “selective reporting” the included articles were classified as low risk. Among the included articles, the article by Tsai et al. (2014) presented the highest index. In contrast, the lowest risk of bias was observed in the article by Olson et al. (2016).

When the article by Bue-Estes et al. (2008) was evaluated (Table 5), it was revealed to be moderate quality with a score of 13 out of a maximum of 24 points (Khan et al., 2015). For the topics “clearly stated aim,” “inclusion of consecutive patients,” “endpoints appropriate to study aim,” “adequate control group,” and “baseline equivalence of groups,” the study achieved the maximum score (2). The article did not report information for the topics “follow-up period appropriates to study aim,” “<5% lost to follow-up,” “prospective calculation of study size” and “contemporary groups,” therefore, these were scored null (0).

**Table 5:** Individual MINORS score.

	**Bue-Estes et al., 2008**
Clearly stated aim	2
Inclusion of consecutive patients	2
Prospective data collection	1
Endpoints appropriate to study aim	2
Unbiased assessment of study endpoint	1
Follow-up period appropriate to study aim	0
< 5% lost to follow-up	0
Prospective calculation of study size	0
Adequate control group	2
Contemporary groups	0
Baseline equivalence of groups	2
Adequate statistical analyses	1
TOTAL	13/24
MINORS Quality score	MQ

## Discussion

This systematic review investigated the effects of moderate physical exercise on cognition. All three studies included in this review (Bue-Estes et al., 2008; Tsai et al., 2014; Olson et al., 2016) concluded that physical exercise seems to improve some cognitive parameters in adults, such as simple reaction time, response accuracy and working memory.

The systematic review could be an effective tool to answer specific questions from primary studies on a subject (Cook et al., 1997), in addition to evaluating the quality of the information and the risk of bias in the articles (Sanderson et al., 2007).

In this systematic review, all studies shown an improvement of cognitive parameters related to moderate physical exercise, such as reduced simple reaction time and improved precision of response to a visual motor stimulus and working memory may be related to exercise intensity (Macpherson et al., 2011), which was measured by VO_2_ max (Bue-Estes et al., 2008; Tsai et al., 2014; Olson et al., 2016). This methodological variable of physical training (intensity) was the same in the three selected articles. In the selected articles, exercise intensity was quantified by the VO_2_ max test, which is considered a gold standard to quantify exercise intensity and provides a basis for ensuring that the obtained results are in accordance by the proposed training.

High-intensity exercise may depress cognitive ability (van den Berg et al., 2016), but moderate-intensity exercise improves this function when the simple reaction time is evaluated (Tsai et al., 2014). On the other hand, studies have shown that sedentary groups who do low-intensity exercise show no difference when compared to sedentary groups (Voss et al., 2011; Olson et al., 2016). The current evident indicates that exercise prescriptions to improve cognition should not be at the extremes of intensity (Voss et al., 2011; Tsai et al., 2014; Olson et al., 2016; van den Berg et al., 2016), and thus should have a linear relationship with this variable, corroborating the findings of this review.

Among the included studies, different forms of exercise performed by individuals from different age groups and both genders, showing similar results on Improvement of cognitive function. This may be related to the characteristics of the exercise performed, which was primarily aerobic in all of the analyzed studies. Quantification of exercise by VO_2_ max (Koutlianos et al., 2013) can be used to individually quantify the intensity according to independent physical exercise variables such as the type of exercise, gender and age.

The cognitive improvements reported in the selected articles may be associated with the capacity of moderate exercise to modulate the central nervous system. Aerobic physical exercise is related to elevated levels of orexin-A and orexin-B (Messina et al., 2016), both neuropeptides synthesized in the hypothalamus that increase neurogenesis and connections between hippocampal neurons, especially in the dentate gyrus, an area involved in cognition (Oomen et al., 2014; Chen et al., 2015; Chieffi et al., 2017a; Trinchero et al., 2017). Orexin-A has neuroprotective and anti-apoptotic effects, and is essential for better performance in terms of attention and working memory (Deadwyler et al., 2007).

Orexin-B/hypocretin-2 (OxB/Hcrt-2) increases the expression of BDNF mRNA leading to increased production of BDNF (Chieffi et al., 2017b), a neurotrophic factor which plays an essential role in cognition (Lee et al., 2015). High levels of BDNF in the hippocampal region modulates long-term potentiation and synaptic plasticity, stimulating learning and memory (Nettiksimmons et al., 2014), in addition to increasing working memory in the prefrontal cortex (Yeom et al., 2016).

Another growth factor that is increased with physical exercise is vascular endothelial gowth factor (VEGF), which promotes angiogenesis (Morland et al., 2017) and is directly associated with neurogenesis and improvement of synaptic function. Thus, VEGF can influence cognitive function through neurogenesis, cerebral blood flow and modulation of long-term potentiation; however, the underlying mechanism has not yet been fully elucidated (Ng et al., 2014).

New blood vessels formed through angiogenesis are unstable (Darland and D'Amore, 1999). The presence of insulin-like growth factor 1 (IGF-1) is essential as it is responsible for the maturation and stability of these neovessels (Jacobo and Kazlauskas, 2015). It is interesting to note that studies have suggested that physical exercise contributes to stabilizing neovessels due to the associated increase in IGF-1 production (Carro et al., 2000; Nakamura et al., 2010; Jacobo and Kazlauskas, 2015).

As suggested by studies and animals and humans, the moderate physical exercise is related to an increase in different neutrophins, as well as to the homeostasis and energetic regulation of nervous system (Kerr et al., 2010; Rhyu et al., 2010; Swain et al., 2012; Marosi and Mattson, 2014; Bathina and Das, 2015; Jacobo and Kazlauskas, 2015; Camerino et al., 2016; Chieffi et al., 2017b,c; Morland et al., 2017). These changes contribute to an increase on plasticity, the formation of new synapses and the integration of neurons to neurons circuits (Gomez-Palacio-Schjetnan and Escobar, 2013; Lu et al., 2014; Phillips, 2017). The improvement of cognition is suggested as a result of this modulation (Colicos and Syed, 2006).

Cognition is a set of processes which functions to classify, recognize and understand information by reasoning through learning and executing responses (Vaynman et al., 2004). However, the three chosen articles only evaluated reaction time parameters, working memory and the accuracy of response. Executive function is an example of a higher cognitive process and includes capabilities such as goal setting, planning and executing action plans and effectively performing actions (Jurado and Rosselli, 2007). Cognition can be modulated throughout life under various stimuli, and varies according to educational level (Liu et al., 2017), nutritional quality (Moody et al., 2017), activity of antioxidants (Farah et al., 2016), and the practice of physical exercise (Atherton et al., 2016; Cadenas-Sanchez et al., 2016).

Several tests are available to the functional evaluation of cognitive parameters (Sanders and Lamers, 2002; Stins et al., 2007; Tsoi et al., 2015). Among them, the primary example of these tests are: STROOP Task (Erdodi et al., 2018), Simon Test (Stoet, 2017), Visual Spatial Attention (Tsai et al., 2016; Byun et al., 2017) and Eriksen Flanker Task (Swatridge et al., 2017; Baumgartner et al., 2018). The Visual Spatial Attention and Eriksen Flanker Task were used among the included studies. All of these tests are used to study the interaction between processing speed, executive functions and working memory, important cognitive domains, which allow us to evaluate selective attention ability and ability, as well as planning, decision making and interference management in real world (Zeischka et al., 2010; Chen et al., 2013; Diamond, 2013).

Another test used in one of the included articles (Bue-Estes et al., 2008) is the Automated Neuropsychological Assessment Metrics (ANAM). This tool is composed by computerized cognitive tests and behavioral questionnaires (Vincent et al., 2017). This tool was previoulsy described as a time-cost efficient feature and presenting a moderate sensitivity and a high specificity when compared to traditional neuropsychological tests,(Jones et al., 2008; Xie et al., 2015; Cole et al., 2017; Paech et al., 2017; Vincent et al., 2017).

In the articles included in this review (Bue-Estes et al., 2008; Tsai et al., 2014; Olson et al., 2016), reaction time as a cognitive parameter was found to be improved, as seen in Table 4. This is an important parameter that can help evaluate the speed of information processing (Batra et al., 2015). These processes were analyzed for their speed and accuracy in terms of response (van Ede et al., 2012), thus evidencing a possible association between moderate physical exercise and improvement of the synaptic network integration involved in this action.

Working memory was another altered cognitive parameter described in one of the articles, which is characterized by the ability to reorganize lists, organize thoughts to form meaningful sentences, incorporate new information, consider options and relate ideas and thoughts (Baddeley, 2012). This was only evaluated in one of the studies (Bue-Estes et al., 2008), in which the authors found an improvement in the group that performed moderate physical exercise compared to the control group and the intensity of the moderate physical exercise group, evidencing a specific effect of exercise intensity.

Cognition can be modulated throughout life under various stimuli, and varies according to educational level (Liu et al., 2017), nutritional quality (Moody et al., 2017), activity of antioxidants (Farah et al., 2016) and the practice of physical exercise (Atherton et al., 2016; Cadenas-Sanchez et al., 2016).

To evaluate the methodological soundness on individual studies regarding their validity and risk of bias, two tools were used: the MINORS tool, a non-randomized tool, and the Cochrane collaboration's tool, used for randomized studies. Among the two randomized studies, one was classified as low risk (Olson et al., 2016) and the other as uncertain risk (Tsai et al., 2014), taking into account the critical key domains chosen for this review.

From the MINORS tool, one of the articles was considered moderate quality (Bue-Estes et al., 2008) while the other had a high risk of bias regarding parameters related to blinding. This was not a critical parameter for this review due to its non-viability, as blinding ensures ignorance (study participants, researchers, medical staff, statisticians) regarding the allocation of participants to one group or another (Higgins et al., 2011).

It is also worth mentioning that during the recruitment of individuals, all the included studies reported that they received information about the procedures and purpose of the research (Bue-Estes et al., 2008; Tsai et al., 2014; Olson et al., 2016). Therefore, this was not one of the major domains for evaluating the quality of the articles included in this systematic review as it interferes with the reliability of this information (Higgins et al., 2011).

## Limitations

Despite the moderate methodological quality and risk of bias of the included articles, moderate physical exercise appears to play a relevant role in the improvement of some cognitive parameters such as simple reaction time, response precision and working memory. However, these are just some of the existing cognitive parameters, and it is important that additional investigations with greater methodological accuracy be carried out by elucidating unexplored parameters in the selected articles.

## Conclusion

Practicing moderate physical exercise may improve cognition in individuals who seek to improve their routinely used cognitive functions and for those who want to prevent or delay cognitive decline.

## Author contributions

RF and MC contributed equally to this work. RF and MC designed the systematic review and supervised the entire program. RL, MdS, and LM reviewed all the studies and extracted the information from the eligible trials. NF and AA analyzed the data and prepared the figures and table. RF and MC wrote the paper. RL, MS, and LM revised the manuscript. All authors reviewed and approved the manuscript.

### Conflict of interest statement

The authors declare that the research was conducted in the absence of any commercial or financial relationships that could be construed as a potential conflict of interest.
