# Supplementary material for: The Effects of Moderate Physical Exercise on Adult Cognition: A Systematic Review
Source: Front Physiol. 2018 Jun 8;9:667. doi: 10.3389/fphys.2018.00667 (PMC6002532; doi:10.3389/fphys.2018.00667)
Supplement: Supplementary file 1 [file Table_1.docx]

Supplementary Material

The effects of moderate physical exercise on adult cognition: a systematic review

Rafael Monteiro Fernandes*, Marcio Gonçalves Correa *, Marcio Antonio Raiol dos Santos, Anna Paula Costa Ponte Sousa Carvalho Almeida, Nathália Carolina Fernandes Fagundes, Lucianne Cople Maia, Rafael Rodrigues Lima^1*^

*** Correspondence:** Rafael Rodrigues Lima; rafalima@ufpa.br

Appendix: Terms used on database search.

| Database | Search format |
| --- | --- |
| PUBMED | ((((((Humans[MeSH Terms]) OR Humans[Title/Abstract]) OR Man, Modern[Title/Abstract]) OR Modern Man[Title/Abstract]) OR Man(Taxonomy)[Title/Abstract]) OR Homo sapiens[Title/Abstract]) OR Human[Title/Abstract])) AND (Adult[MeSH Terms]) OR Adult[Title/Abstract]) OR Adults[Title/Abstract])) AND (((((((((((Cognition[MeSH Terms]) OR Cognition[Title/Abstract]) OR Cognitions) OR Cognitive Function) OR Cognitive Functions) OR Function, Cognitive) OR Functions, Cognitive)) OR ((((((((Awareness[MeSH Terms]) OR Awareness[Title/Abstract]) OR Situational Awareness[Title/Abstract]) OR Awarenesses[Title/Abstract]) OR Awareness, Situational[Title/Abstract]) OR Awarenesses, Situational[Title/Abstract]) OR Situational Awarenesses[Title/Abstract]) OR Situation Awareness[Title/Abstract]) OR Awareness, Situation[Title/Abstract] OR Awarenesses, Situation[Title/Abstract] OR Situation, Awarenesses[Title/Abstract])) OR (((((Cognitive Dissonance[MeSH Terms]) OR Cognitive Dissonances[Title/Abstract]) OR Dissonance, Cognitive[Title/Abstract]) OR Dissonances, Cognitive[Title/Abstract]) OR Cognitive Dissonance[Title/Abstract])) OR (((((((((((((((Cognitive Reserve[MeSH Terms]) OR Cognitive Reserve[Title/Abstract]) OR Cognitive Reserves[Title/Abstract]) OR Reserve, Cognitive[Title/Abstract]) OR Reserves, Cognitive[Title/Abstract]) OR Brain Reserve[Title/Abstract]) OR Brain Reserve[Title/Abstract]) OR Reserve, Brain[Title/Abstract]) OR Reserves, Brain[Title/Abstract]) OR Comprehension[MeSH Terms]) OR Comprehension[Title/Abstract]) OR Comprehension Readability[Title/Abstract]) OR Consciousness[MeSH Terms]) OR Consciousness[Title/Abstract]) OR Consciousnesses[Title/Abstract]))) NOT ((Aged[MeSH Terms]) OR Aged[Title/Abstract]) OR Elderly[Title/Abstract]) OR Nervous System Diseases[MeSH Terms]) OR Nervous System Diseases[Title/Abstract]) OR Disease, Nervous System[Title/Abstract]) OR Diseases, Nervous System[Title/Abstract]) OR Nervous System Disease[Title/Abstract]) OR Neurologic Disorders[Title/Abstract]) OR Disorder, Neurologic[Title/Abstract]) OR Disorders, Neurologic[Title/Abstract]) OR Neurologic Disorder[Title/Abstract]) OR Neurological Disorders[Title/Abstract]) OR Disorder, Neurological[Title/Abstract]) OR Disorders, Neurological[Title/Abstract]) OR Neurological Disorder[Title/Abstract]) OR Nervous System Disorders[Title/Abstract]) OR Disorder, Nervous System[Title/Abstract]) OR Disorders, Nervous System[Title/Abstract]) OR Nervous System Disorder[Title/Abstract])) AND (Motor Activity[MeSH Terms]) OR Motor Activity[Title/Abstract]) OR Activities, Motor[Title/Abstract]) OR Activity, Motor[Title/Abstract]) OR Motor Activities[Title/Abstract]) OR Physical Activity[Title/Abstract]) OR Activities, Physical[Title/Abstract]) OR Activity, Physical[Title/Abstract]) OR Physical Activities[Title/Abstract]) OR Locomotor Activity[Title/Abstract]) OR Activities, Locomotor[Title/Abstract]) OR Activity, Locomotor[Title/Abstract]) OR Locomotor Activities[Title/Abstract]) OR Exercise[MeSH Terms]) OR Exercise[Title/Abstract]) OR Exercise, Physical[Title/Abstract]) OR Exercises[Title/Abstract]) OR Exercises, Physical[Title/Abstract]) OR Physical Exercise[Title/Abstract]) OR Physical Exercises[Title/Abstract]) OR Exercise, Isometric[Title/Abstract]) OR Exercises, Isometric[Title/Abstract]) OR Isometric Exercises[Title/Abstract]) OR Isometric Exercise[Title/Abstract]) OR Exercise, Aerobic[Title/Abstract]) OR Aerobic Exercises[Title/Abstract]) OR Exercises, Aerobic[Title/Abstract]) OR Aerobic Exercise[Title/Abstract]) OR Physical Fitness[MeSH Terms]) OR Physical Fitness[Title/Abstract]) OR Fitness, Physical[Title/Abstract]) |
| SCOPUS | ( TITLE-ABS-KEY ( "Motor Activity" ) OR TITLE-ABS-KEY ( " Activities, Motor" ) OR TITLE-ABS-KEY ( "Activity, Motor" ) OR TITLE-ABS-KEY ( "Motor Activities" ) OR TITLE-ABS-KEY ( "Physical Activity" ) OR TITLE-ABS-KEY ( "Activities, Physical" ) OR TITLE-ABS-KEY ( "Activity, Physical" ) OR TITLE-ABS-KEY ( " Physical Activities" ) OR TITLE-ABS-KEY ( "Locomotor Activity" ) OR TITLE-ABS-KEY ( "Activities, Locomotor" ) OR TITLE-ABS-KEY ( "Activity, Locomotor" ) OR TITLE-ABS-KEY ( "Locomotor Activities" ) OR TITLE-ABS-KEY ( "Exercise" ) OR TITLE-ABS-KEY ( " Exercises" ) OR TITLE-ABS-KEY ( "Exercise, Physical" ) OR TITLE-ABS-KEY ( "Exercises, Physical" ) OR TITLE-ABS-KEY ( "Physical Exercise" ) OR TITLE-ABS-KEY ( "Physical Exercises" ) OR TITLE-ABS-KEY ( "Exercise, Isometric" ) OR TITLE-ABS-KEY ( "Exercises, Isometric" ) OR TITLE-ABS-KEY ( "Isometric Exercises" ) OR TITLE-ABS-KEY ( "Isometric Exercise" ) OR TITLE-ABS-KEY ( "Exercise, Aerobic" ) OR TITLE-ABS-KEY ( "Aerobic Exercises" ) OR TITLE-ABS-KEY ( "Exercises, Aerobic" ) OR TITLE-ABS-KEY ( "Aerobic Exercise" ) OR TITLE-ABS-KEY ( "Physical Fitness" ) OR TITLE-ABS-KEY ( "Fitness, Physical" ) ) AND ( ( TITLE-ABS-KEY ( humans ) OR TITLE-ABS-KEY ( "Man, Modern" ) OR TITLE-ABS-KEY ( "Modern Man" ) OR TITLE-ABS-KEY ( "Man Taxonomy" ) OR TITLE-ABS-KEY ( "Homo sapiens" ) OR TITLE-ABS-KEY ( human ) ) AND ( TITLE-ABS-KEY ( adult ) OR TITLE-ABS-KEY ( adults ) ) AND ( TITLE-ABS-KEY ( cognition ) OR TITLE-ABS-KEY ( cognitions ) OR TITLE-ABS-KEY ( "Cognitive Function" ) OR TITLE-ABS-KEY ( "Cognitive Functions" ) OR TITLE-ABS-KEY ( "Function, Cognitive" ) OR TITLE-ABS-KEY ( "Functions, Cognitive" ) OR TITLE-ABS-KEY ( awareness ) OR TITLE-ABS-KEY ( awarenesses ) OR TITLE-ABS-KEY ( "Situational Awareness" ) OR TITLE-ABS-KEY ( "Awareness, Situational" ) OR TITLE-ABS-KEY ( "Awarenesses, Situational" ) OR TITLE-ABS-KEY ( "Situational Awarenesses" ) OR TITLE-ABS-KEY ( "Situation Awareness" ) OR TITLE-ABS-KEY ( "Awareness, Situation" ) OR TITLE-ABS-KEY ( "Awarenesses, Situation" ) OR TITLE-ABS-KEY ( situation awarenesses ) OR TITLE-ABS-KEY ( "Cognitive Dissonance" ) OR TITLE-ABS-KEY ( "Cognitive Dissonances" ) OR TITLE-ABS-KEY ( "Dissonance, Cognitive" ) OR TITLE-ABS-KEY ( "Dissonances, Cognitive" ) OR TITLE-ABS-KEY ( "Cognitive Reserves" ) OR TITLE-ABS-KEY ( "Reserve, Cognitive" ) OR TITLE-ABS-KEY ( "Reserves, Cognitive" ) OR TITLE-ABS-KEY ( "Brain Reserve" ) OR TITLE-ABS-KEY ( "Brain Reserves" ) OR TITLE-ABS-KEY ( "Reserve, Brain" ) OR TITLE-ABS-KEY ( "Reserves, Brain" ) OR TITLE-ABS-KEY ( comprehension ) OR TITLE-ABS-KEY ( "Comprehension Readability" ) OR TITLE-ABS-KEY ( consciousness ) OR TITLE-ABS-KEY ( consciousnesses ) ) AND NOT ( TITLE-ABS-KEY ( aged ) OR TITLE-ABS-KEY ( elderly ) OR TITLE-ABS-KEY ( "Nervous System Diseases" ) OR TITLE-ABS-KEY ( "Disease, Nervous System" ) OR TITLE-ABS-KEY ( "Diseases, Nervous System" ) OR TITLE-ABS-KEY ( "Nervous System Disease" ) OR TITLE-ABS-KEY ( "Neurologic Disorders" ) OR TITLE-ABS-KEY ( "Disorder, Neurologic" ) OR TITLE-ABS-KEY ( "Disorders, Neurologic" ) OR TITLE-ABS-KEY ( "Neurologic Disorder" ) OR TITLE-ABS-KEY ( "Neurological Disorders" ) OR TITLE-ABS-KEY ( "Disorder, Neurological" ) OR TITLE-ABS-KEY ( "Disorders, Neurological" ) OR TITLE-ABS-KEY ( "Neurological Disorder" ) OR TITLE-ABS-KEY ( "Nervous System Disorders" ) OR TITLE-ABS-KEY ( " Disorder, Nervous System" ) OR TITLE-ABS-KEY ( "Disorders, Nervous System" ) OR TITLE-ABS-KEY ( "Nervous System Disorder" ) ) ) |
| COCHRANE | Humans or Man, Modern or Modern Man or Man (Taxonomy) or Homo sapiens or Human AND Adult or Adults AND Cognition or Cognitions or Cognitive Function or Cognitive Functions or Function, Cognitive or Functions, Cognitive or Awareness or Awarenesses or Situational Awareness or Awareness, Situational or Awarenesses, Situational or Situational Awarenesses or Situation Awareness or Awareness, Situation or Awarenesses, Situation or Situation Awarenesses or Cognitive Reserve or Cognitive Reserves or Reserve, Cognitive or Reserves, Cognitive or Brain Reserve or Brain Reserves or Reserve, Brain or Reserves, Brain or Comprehension or Comprehension Readability or Consciousness or Consciousnesses NOT Aged or Elderly or Nervous System Diseases or Disease, Nervous System or Diseases, Nervous System or Nervous System Disease or Neurologic Disorders or Disorder, Neurologic or Disorders, Neurologic or Neurologic Disorder or Neurological Disorders or Disorder, Neurological or Disorders, Neurological or Neurological Disorder or Nervous System Disorders or Disorder, Nervous System or Disorders, Nervous System or Nervous System Disorder AND Motor Activity or Activities, Motor or Activity, Motor or Motor Activities or Physical Activity or Activities, Physical or Activity, Physical or Physical Activities or Locomotor Activity or Activities, Locomotor or Activity, Locomotor or Locomotor Activities or Exercise or Exercises or Exercise, Physical or Exercises, Physical or Physical Exercise or Physical Exercises or Exercise, Isometric or Exercises, Isometric or Isometric Exercises or Isometric Exercise or Exercise, Aerobic or Aerobic Exercises or Exercises, Aerobic or Aerobic Exercise or Physical Fitness or Fitness, Physical |
| WEB OF SCIENCE | TS=(Humans) OR TS=(Man, Modern) OR TS=(Modern Man) OR TS=(Man Taxonomy) OR TS=(Homo sapiens) OR TS=(Human) **AND** TS=(Adult) OR TS=(Adults) **AND** TS=(Cognition) OR TS=(Cognitions) OR TS=(Cognitive Function) OR TS=(Cognitive Functions) OR TS=(Function, Cognitive) OR TS=(Functions, Cognitive) OR TS=(Awareness) OR TS=( Awarenesses) OR TS=(Situational Awareness) OR TS=(Awareness, Situational) OR TS=(Awarenesses, Situational) OR TS=(Situational Awarenesses) OR TS=(Situation Awareness) OR TS=(Awareness, Situation) OR TS=(Awarenesses, Situation) OR TS=(Situation Awarenesses)OR TS=(Cognitive Dissonance) OR TS=( Cognitive Dissonances) OR TS=(Dissonance, Cognitive) OR TS=(Dissonances, Cognitive) OR TS=(Cognitive Reserve) OR TS=(Cognitive Reserves) OR TS=(Reserve, Cognitive) OR TS=(Reserves, Cognitive) OR TS=(Brain Reserve) OR TS=(Brain Reserves) OR TS=(Reserve, Brain) OR TS=(Reserves, Brain)OR TS=(Comprehension) OR TS=(Comprehension Readability)OR TS=(Consciousness) OR TS=( Consciousnesses) **AND NOT** TS=(Aged) OR TS=( Elderly)OR TS=(Nervous System Diseases) OR TS=( Disease, Nervous System) OR TS=(Diseases, Nervous System) OR TS=(Nervous System Disease) OR TS=(Neurologic Disorders) OR TS=(Disorder, Neurologic) OR TS=(Disorders, Neurologic) OR TS=(Neurologic Disorder) OR TS=(Neurological Disorders) OR TS=(Disorder, Neurological) OR TS=(Disorders, Neurological) OR TS=(Neurological Disorder) OR TS=(Nervous System Disorders) OR TS=(Disorder, Nervous System) OR TS=(Disorders, Nervous System) OR TS=(Nervous System Disorder) **AND** TS=(Motor Activity) OR TS=(Activities, Motor) OR TS=(Activity, Motor) OR TS=(Motor Activities) OR TS=(Physical Activity) OR TS=(Activities, Physical) OR TS=(Activity, Physical) OR TS=(Physical Activities) OR TS=(Locomotor Activity) OR TS=(Activities, Locomotor) OR TS=(Activity, Locomotor) OR TS=(Locomotor Activities) OR TS=(Exercise) OR TS=(Exercises) OR TS=(Exercise, Physical) OR TS=(Exercises, Physical) OR TS=(Physical Exercise) OR TS=(Physical Exercises) OR TS=(Exercise, Isometric) OR TS=(Exercises, Isometric) OR TS=(Isometric Exercises) OR TS=(Isometric Exercise) OR TS=(Exercise, Aerobic) OR TS=(Aerobic Exercises) OR TS=(Exercises, Aerobic) OR TS=(Aerobic Exercise) OR TS=(Physical Fitness) OR TS=(Fitness, Physical) |
| OPENGREY | " Cognition OR Awareness AND Exercise |
| LILACS | (tw:Humans)))) OR (tw:((tw:(Man, Modern)))) OR (tw:((tw:(Modern Man)))) OR (tw:((tw:(Man (Taxonomy))) OR (tw:(Homo sapiens)) OR (tw:(Human)) AND (tw:(Adult)) OR (tw:(Adults)) AND (tw:(Cognition)) OR (tw:(Cognitions)) OR (tw:(Cognitive Function)) OR (tw:(Cognitive Functions)) OR (tw:(Function, Cognitive)) OR (tw:(Functions, Cognitive)) OR (tw:(Awareness)) OR (tw:(Awarenesses)) OR (tw:(Situational Awareness)) OR (tw:(Awareness, Situational)) OR (tw:(Awarenesses, Situational)) OR (tw:(Situational Awarenesses)) OR (tw:(Situation Awareness)) OR (tw:(Awareness, Situation)) OR (tw:(Awarenesses, Situation)) OR (tw:(Situation Awarenesses)) OR (tw:(Cognitive Reserve)) OR (tw:(Cognitive Reserves)) OR (tw:(Reserve, Cognitive)) OR (tw:(Reserves, Cognitive)) OR (tw:(Brain Reserve)) OR (tw:(Brain Reserves)) OR (tw:(Reserve, Brain)) OR (tw:(Reserves, Brain)) OR (tw:(Comprehension)) OR (tw:(Comprehension Readability)) OR (tw:(Consciousness)) OR (tw:(Consciousnesses)) AND NOT (tw:( Aged)) OR (tw:(Elderly)) OR (tw:(Nervous System Diseases)) OR (tw:(Disease, Nervous System)) OR (tw:(Diseases, Nervous System)) OR (tw:(Nervous System Disease)) OR (tw:(Neurologic Disorders)) OR (tw:(Disorder, Neurologic)) OR (tw:(Disorders, Neurologic)) OR (tw:(Neurologic Disorder)) OR (tw:(Neurological Disorders)) OR (tw:(Disorder, Neurological)) OR (tw:(Disorders, Neurological)) OR (tw:(Neurological Disorder)) OR (tw:(Nervous System Disorders)) OR (tw:(Disorder, Nervous System)) OR (tw:(Disorders, Nervous System)) OR (tw:(Nervous System Disorder)) AND (tw:(Motor Activity)) OR (tw:(Activities, Motor)) OR (tw:(Activity, Motor)) OR (tw:(Motor Activities)) OR (tw:(Physical Activity)) OR (tw:(Activities, Physical)) OR (tw:(Activity, Physical)) OR (tw:(Physical Activities)) OR (tw:(Locomotor Activity)) OR (tw:(Activities, Locomotor)) OR (tw:(Activity, Locomotor)) OR (tw:(Locomotor Activities)) OR (tw:(Exercise)) OR (tw:(Exercises)) OR (tw:(Exercise, Physical)) OR (tw:(Exercises, Physical)) OR (tw:(Physical Exercise)) OR (tw:(Physical Exercises)) OR (tw:(Exercise, Isometric)) OR (tw:(Exercises, Isometric)) OR (tw:(Isometric Exercises)) OR (tw:(Isometric Exercise)) OR (tw:(Exercise, Aerobic)) OR (tw:(Aerobic Exercises)) OR (tw:(Exercises, Aerobic)) OR (tw:(Aerobic Exercise)) OR (tw:(Physical Fitness)) OR (tw:(Fitness, Physical)) |
| GOOGLE SCHOLAR | Consciousness AND Comprehension AND Cognitive Reserve AND Cognitive Dissonance AND Awareness AND Adult AND Humans AND Cognition AND Exercise AND Motor Activity AND Physical Fitness NOT Nervous System Diseases NOT Aged NOT Elderly |
| CENTRAL | Humans or Man, Modern or Modern Man or Man (Taxonomy) or Homo sapiens or Human **AND** Adult or Adults **AND** Cognition or Cognitions or Cognitive Function or Cognitive Functions or Function, Cognitive or Functions, Cognitive or Awareness or Awarenesses or Situational Awareness or Awareness, Situational or Awarenesses, Situational or Situational Awarenesses or Situation Awareness or Awareness, Situation or Awarenesses, Situation or Situation Awarenesses or Cognitive Reserve or Cognitive Reserves or Reserve, Cognitive or Reserves, Cognitive or Brain Reserve or Brain Reserves or Reserve, Brain or Reserves, Brain or Comprehension or Comprehension Readability or Consciousness or Consciousnesses **NOT** Aged or Elderly or Nervous System Diseases or Disease, Nervous System or Diseases, Nervous System or Nervous System Disease or Neurologic Disorders or Disorder, Neurologic or Disorders, Neurologic or Neurologic Disorder or Neurological Disorders or Disorder, Neurological or Disorders, Neurological or Neurological Disorder or Nervous System Disorders or Disorder, Nervous System or Disorders, Nervous System or Nervous System Disorder **AND** Motor Activity or Activities, Motor or Activity, Motor or Motor Activities or Physical Activity or Activities, Physical or Activity, Physical or Physical Activities or Locomotor Activity or Activities, Locomotor or Activity, Locomotor or Locomotor Activities or Exercise or Exercises or Exercise, Physical or Exercises, Physical or Physical Exercise or Physical Exercises or Exercise, Isometric or Exercises, Isometric or Isometric Exercises or Isometric Exercise or Exercise, Aerobic or Aerobic Exercises or Exercises, Aerobic or Aerobic Exercise or Physical Fitness or Fitness, Physical |
